# Women’s outcomes following mixed-sex, women-only, and home-based cardiac rehabilitation participation and comparison by sex

**DOI:** 10.1186/s12905-021-01553-5

**Published:** 2021-12-15

**Authors:** Fiorella A. Heald, Susan Marzolini, Tracey J. F. Colella, Paul Oh, Rajni Nijhawan, Sherry L. Grace

**Affiliations:** 1 Faculty of Health, York University, Bethune 368, 4700 Keele Street, Toronto, ON M3J 1P3 Canada; 2 KITE-Toronto Rehabilitation Institute, University Health Network, University of Toronto, Toronto, ON Canada

**Keywords:** Cardiac rehabilitation, Coronary heart disease, Outcomes, Quality of life, Women, Sex differences

## Abstract

**Background:**

Despite women’s greater need for cardiac rehabilitation (CR), they are less likely to utilize it. Innovative CR models have been developed to better meet women’s needs, yet there is little controlled, comparative data assessing the effects of these models for women. This study compared outcomes in women electing to participate in mixed-sex, women-only, or home-based CR, and a matched sample of men.

**Methods:**

In this retrospective study, electronic records of CR participants in Toronto who were offered the choice of program model between January 2017 and July 2019 were analyzed; clinical outcomes comprised cardiorespiratory fitness, risk factors and psychosocial well-being. These were assessed at intake and post-6-month program and analyzed using general linear mixed models.

**Results:**

There were 1181 patients (727 women [74.7% mixed, 22.0% women-only, 3.3% home-based]; 454 age and diagnosis-matched men) who initiated CR; Cardiorespiratory fitness among women was higher at initiation of mixed-sex than women-only (METs 5.1 ± 1.5 vs 4.6 ± 1.3; *P* = .007), but no other outcome differences were observed. 428 (58.9%) women completed the programs, with few women retained in the home-based model limiting comparisons. There were significant improvements in high-density lipoprotein cholesterol (*P* = .001) and quality of life (*P* = .001), and lower depressive symptoms (*P* = .030) as well as waist circumference (*P* = .001) with mixed-sex only. VO_2peak_ was significantly higher at discharge in mixed-sex than women-only (estimate = 1.67, standard error = 0.63, 95% confidence interval = 0.43–2.91).

**Conclusion:**

Participation in non-gender-tailored women-only CR was not advantageous as expected. More research is needed, particularly including women participating in home-based programs.

## Introduction

Cardiovascular Disease (CVD) is the leading cause of morbidity (13.5% of total disability-adjusted life years) and mortality (33% of total deaths) for women globally [1]. Furthermore, women with CVD experience worse outcomes than men [2], with higher mortality rates following myocardial infarction, percutaneous coronary intervention (PCI), and coronary artery bypass graft (CABG) surgery [3, 4]. With regard to morbidity, women with acute coronary syndrome and those after coronary revascularization have longer hospitalizations and higher in-hospital mortality, and have 30% more readmissions within 30 days after the index hospitalization compared to men [4].

Cardiac rehabilitation (CR) is an outpatient, comprehensive model of care for secondary prevention, which can mitigate the above burden. These programs are generally offered in clinical centres under supervision. CR has been shown to improve outcomes, including quality of life [5], hospital readmission rates, revascularization rates [6], and mortality [7]. While there are little randomized data on women’s CR outcomes specifically [8], observational data suggests women may have even lower mortality than men where they fully participate [9, 10]. Clearly, women are in great need of these services given their poorer cardiovascular outcomes, outlined above. Given the observational data on the benefits in women as well [11–13], the clinical practice guidelines for women with CVD recommend referral to CR [14].

However, CR utilization is sub-optimal [15], and even lower in women [16–18]. Women’s CR barriers have been extensively studied [19, 20], and women-focused models have been developed to address them [21, 22]. These are programs where: (1) some or all components or sessions, (2) comprise all or mostly women (and staff), and/or (3) content may be tailored to meet women’s unique needs and preferences [23]. Moreover, home-based models (i.e., patients are supported remotely in their risk reduction) [24] may overcome women’s common barriers such as transportation and time constraints due to family role obligations, and indeed some women prefer this approach [25, 26]. Equivalent outcomes are observed with home-based and supervised programs, however most participants in the Cochrane review were men [27].

There are a limited number of studies regarding women-focused CR that include comparison groups, and even fewer comparing women in all 3 models, and to men [28]. Therefore, the objectives of this study were to compare: (1) cardiorespiratory fitness, (2) risk factors (i.e., blood pressure, lipids, tobacco use, and anthropometrics), and (3) psychosocial well-being (i.e., depressive symptoms and quality of life), in women between the three CR models. These outcomes are described in a matched sample of men in supervised CR for comparison purposes.

## Methods

### Design and procedure

This was a retrospective cohort study, with 4 comparison groups. Data used in this study were extracted from an electronic patient management record utilized across the University Health Network (UHN) Cardiovascular Prevention and Rehabilitation sites located in Toronto, Ontario, Canada, from January 1, 2017 to February 28, 2020 (only to March 31, 2019 for Toronto Western site as the program was shortened to 4 months at that time due to the wait list); data were extracted from pre and post-program.

### Setting

UHN is an academic health sciences center comprised of several hospitals, with an advanced cardiac program. There are 2 CR programs (at Toronto Western Hospital [acute care centre] and Toronto Rehabilitation Institute [large outpatient centre]) and 1 satellite at a local university; data from the satellite were not included as many participants are stroke patients and the program is of shorter duration. The median wait time to start the programs is 42 days [29]. Staff at both centers are multidisciplinary, with extensive experience.

Before starting the program, every patient completes an intake assessment, including questionnaires (e.g., depressive symptom screens, medical history, health behaviours), risk factor assessment (e.g., body composition), a consultation with a program physician (e.g., medication review). A follow-up appointment for an exercise stress test is scheduled, most commonly on a treadmill using a modified Bruce protocol. All data are entered into the electronic record. Patients are reassessed at the end of their program (after 6 months), inclusive of risk factor assessment and functional capacity testing.

#### Models

At both centres, at the time of the exercise stress test, patients are given the option to choose between the supervised program at the centres or the home-based program, and women have the additional option of the supervised women-only program. Model selection is primarily based on patient preference rather than clinical criteria, although patients are encouraged to enroll in a class that best suits their medical condition.

Also at both centres, both the supervised mixed-sex and women-only models offer a comprehensive program that consists of structured exercise, patient education, risk factor management, dietary as well as psychosocial counselling, in addition to other components as needed. After the intake assessment, there are weekly classes on-site over 6 months (25 sessions total); each class lasts approximately 90 min.

Aerobic exercise is individually prescribed, to be performed 5 days per week. Initial training intensity is based on patients’ exercise stress test, in accordance with the American College of Sports Medicine guidelines [30]. Target heart rate is calculated using the anaerobic threshold and heart rate reserve, with an exercise intensity range of 60% to 80%; the intensity of exercise may be adjusted to achieve an 11–15 rating of perceived exertion [31]. During CR, participants walk/jog on the track (Toronto Rehab only) or use treadmills and bicycles. Prescription progression is considered approximately every 2 weeks as exercise specialists deem fit, with the goal of increasing to 30–60 min per session. As most patients are on a beta-blocker, exercise intensity is monitored using perceived exertion (heart rate used as a guideline).

Patients are prescribed resistance training unless contraindicated or limited by comorbidities. Resistance training is prescribed 2–3 sessions per week, comprising 7–10 upper and lower body exercises. Initial weight loads of 60% of 1-repetition maximum are used; one set of 10 repetitions is prescribed initially and patients progress to 2 sets and increase to 15 repetitions before weights increase [32]. Handheld dumbbells, resistance bands, and/or body weight are used. All patients are encouraged to document their exercise in an exercise diary that is reviewed by the exercise specialists weekly.

The patient education program is evidence-based, and delivered in a group setting in conjunction with each visit (https://www.healtheuniversity.ca/en/cardiaccollege/Pages/default.aspx) [33]; patients are encouraged to read the corresponding booklet in advance and bring it to each session, where the patients are engaged in the education through adult learning principles.

Participants are screened for depressive symptoms, with those screening positive being referred to the program social worker or psychologist. All participants are offered a group stress management program, and 1–1 sessions with the registered dietitian. Aside from women being the only sex participating in the women-only program and that examples during patient education are tailored to the audience, all aspects of the program are consistent with supervised mixed-sex program (i.e., not gender-tailored content).

The home-based model involves a personalized program to be followed at home [34], supported by online patient education. In addition to the on-site assessments as outlined above, home-based patients also come to the centre for a one-on-one orientation, as well as an aerobic exercise trial and resistance training instruction at the start of the program. Patients have weekly telephone consultation for the first 3 months, and less frequent telephone consultation thereafter through month 6 (same overall program duration as supervised models), each lasting for 15 min on average, for a total average of 15 calls. Patients come on-site for stress management and/or 1–1 dietary counselling as per their needs, and for their exit assessment as in the supervised programs (i.e., program completion). Otherwise, exercise prescription and progression as well as other components are synonymous with the supervised models and across the two centres.

### Participants

To be included in the program, patients had to be 18 years or older with at least one of the following indications: coronary artery disease (CAD)/acute coronary syndrome, spontaneous coronary artery dissection, atrial fibrillation, adult congenital heart disease, cardiomyopathy, following cardiac interventions (i.e., PCI, CABG, valve intervention/surgery, implantable rhythm device, aneurysm repair, and ablation), and those at risk for developing CAD or CVD (minimum of 3 modifiable cardiac risk factors, including diabetes, hypertension, dyslipidemia, current/recent tobacco use, depression, family history).

The program has separate classes for patients with stroke, heart failure, heart transplant, diabetes, and breast cancer, which were not considered in this study. Those who had exercise-limiting medical issues such as pulmonary disease, were at significant risk of a fall, and with significant cognitive and/or uncontrolled serious mental health (i.e., not anxiety, etc.) issues which would impede safe participation were excluded from the program.

Study-specific inclusion criteria were graduated female patients who attended at least 1 on-site exercise session for supervised models and 1 telephone consultation for the home-based model; males who were in the supervised model were eligible for matching. Patients were considered to have completed the program if they did not fail to attend 2–3 consecutive CR sessions (telephone consultations for home-based model) without notice and respond to communication attempts made by the program, and completed the post-program assessments [35].

### Measures

Sociodemographic (e.g., age at enrollment, marital status, language spoken, highest educational attainment, occupational status, travel time to CR centre; data on ethnocultural background were not available) and some clinical characteristics (e.g., tobacco use: current, former/never) were obtained from questionnaires that patients completed prior to program initiation. Clinical data were also extracted from the referral form (e.g., cardiac event/procedure) and initial assessments.

#### Outcomes

Outcomes were measured at intake and discharge assessments. Cardiorespiratory fitness was operationalized as VO_2peak_ (volume of oxygen consumed per unit of time [30], expressed in mL/kg/min; Toronto Rehab) or metabolic equivalent of task (METs; Toronto Western), which were obtained during the graded exercise stress test. Given the measurement error associated with the latter, while results from the Toronto Rehab cardiopulmonary assessments were converted to METs so results across both sites could be grouped, VO_2peak_ was also extracted and compared in the Toronto Rehab participants.

With regard to risk factors, systolic blood pressure (SBP) and diastolic blood pressure (DBP), measured in mmHg, were assessed at Toronto Rehab using microphone-assisted Korotkoff auscultation by a cardiopulmonary technologist or attending physician prior to the cardiopulmonary exercise test (position depending on the exercise test modality). At Toronto Western, BP was assessed pre and post via manual sphygmomanometer. The initial BP target in adults is < 140 mmHg for SBP and < 90 mmHg for DBP [36, 37].

Lipid profile was recorded from bloodwork results from patient’s referral information or standard medical laboratory report where available (not all patients went for the test despite provision of requisition post-program). The American College of Cardiology recommends a low-density lipoprotein cholesterol (LDL-C) goal of 1.8 mmol/L for very high-risk patients, such as those in the current cohort with a history of multiple major atherosclerotic CVD events or 1 major atherosclerotic CVD event and multiple high-risk conditions [38]. Waist circumference measurements (in centimeters) were taken horizontally around the abdomen at the narrowest part of the torso between the iliac crest and the xiphoid process (or at the level of the iliac crest if narrowest part is not available) at end expiration in standing position using a tape measure; a cut-off of ≥ 88 cm in women and ≥ 102 cm in men [38] was used as target. Body mass index was defined as body mass, measured using the InBody 520 body composition analyzer (Biospace Co., Ltd., Seoul, South Korea), divided by the square of body height (kg/m^2^; stadiometer). Tobacco use is outlined above.

In terms of psychosocial well-being, depressive symptoms were self-reported using the 20-item validated Centre for Epidemiologic Studies-Depression (CES-D) scale (only administered at Toronto Rehab) [39]. Where 4 or less item scores were missing, the mean score on completed items for that participant was used; where more items were not completed the data were not used. Scores range from 0 to 60; higher scores indicate greater depressive symptoms, and scores ≥ 16 indicate suspected depression. Quality of life was measured with Cantril’s ladder [40]. Patients are presented with a picture of a ladder with steps numbered from 0 at the bottom, representing the worst possible life, to 10 at the top, representing the best possible life for them. Patients were asked on which rung of the ladder they felt they personally stood at the present time.

### Statistical analysis

Statistical analyses were performed using IBM SPSS Statistics for Macintosh, version 26.0, with statistical significance defined as *P* < 0.05.

First, descriptive statistics were used to describe the characteristics of participants at CR intake, by model. Results were reported as numbers and percentages (%) for categorical variables and mean ± standard deviation (SD) for continuous variables. Variables were scrutinized to determine whether they were normally distributed; non-parametric tests were applied where they were not, as outlined below.

For the sex comparisons, women were first matched to men based on age (1–2 years interval) and cardiac intervention (CABG and non-CABG) using the Case–Control Matching procedure without replacement. Then, differences in any sociodemographic and clinical characteristics at intake as well as wait time between matched men and women in the mixed-sex model were compared using the Mann–Whitney U test and chi-square tests, as appropriate (age and sex were different before matching, with women older and less often having CABG).

Pre-CR sociodemographic and clinical characteristics of women retained in the program versus lost to follow-up (i.e., did not complete any post-program assessments) were compared using the Mann–Whitney U test or chi-square as applicable. Then, within-subject outcome changes from intake to discharge in each CR model (women and men) among completers were explored using paired-samples t-test for continuous measures, and McNemar’s test for analysis of tobacco use. Finally, to assess between-group changes, for continuous outcomes, general linear mixed models were performed with the clinical outcome as the dependent variable, CR model (or sex in the women and men comparison) and timepoint as fixed effects parameters, and intercept as random effects parameter; the women-only model and men were used as reference categories. Tobacco use could not be compared between CR models due to low numbers.

## Results

### Cohort characteristics

The female cohort during the period of study comprised 543 participants in the supervised mixed-sex, 160 in supervised women-only, and 24 in the home-based model; model choice is considered elsewhere [41]. The matched cohort comprised 454 women and 454 men in supervised mixed-sex; thus, there were 1181 participants in total. Their sociodemographic and clinical characteristics are shown in Table 1; other referral indications were primarily aneurysm, congenital heart disease, and heart transplant. Ninety-nine (14.2%) women had elevated SBP, 39 (5.6%) elevated DBP, 264 (48.4%) LDL-C above target, 390 (55.9%) waist circumference above target, and 152 (36.9%) elevated depressive symptoms at baseline.

**Table 1:** Participant’s sociodemographic and clinical characteristics pre-program by model and sex

	Men*	Women†			
	Mixed-sex N = 454	Mixed-sex N = 543 (74.7%)	Women-only N = 160 (22.0%)	Home-based N = 24 (3.3%)	Total N = 727
*Sociodemographic*					
Age	66.8 ± 11.8	66.7 ± 12.1	68.1 ± 12.1	61.3 ± 16.9	66.9 ± 12.3
Marital status (% married/common-law)	290 (82.2%)***	257 (67.8%)	67 (63.2%)	9 (60.0%)	333 (66.6%)
Highest educational attainment (% ≥ high school)	226 (93.8%)	299 (92.3%)	79 (94.0%)	12 (100.0%)	390 (92.9%)
Language spoken (% English)	313 (95.1%)	411 (96.3%)	111 (98.2%)	20 (100.0%)	542 (96.8%)
Occupational status					
Retired/no formal employment	147 (54.9%)	220 (64.1%)‡‡	74 (80.4%)‡‡	6 (54.5%)	300 (67.3%)†
Full-time/part-time/ modified/restricted duties	101 (37.7%)	103 (30.0%)‡	16 (17.4%)‡	4 (36.4%)	123 (27.6%)†
Other (e.g., disability)	20 (7.5%)	20 (5.8%)	2 (2.2%)	1 (9.1%)	23 (5.2%)
Travel time to CR centre (% 0–30 min)	124 (63.3%)	143 (61.1%)	34 (58.6%)	2 (40.0%)	179 (60.3%)
Living situation					
With spouse/partner	212 (70.2%)***	201 (50.9%)	44 (43.6%)	11 (68.8%)	256 (50.0%)
Alone	41 (13.6%)***	123 (31.1%)	37 (36.6%)	4 (25.0%)	164 (32.0%)
With family/friends/others	49 (16.2%)	71 (18.0%)	20 (19.8%)	1 (6.3%)	92 (18.0%)
*Clinical characteristics*					
Referral event/procedure§					
PCI	228 (50.6%)***	185 (34.4%)	54 (34.0%)	7 (31.8%)	246 (34.2%)
Valvular heart disease	34 (7.5%)*	62 (11.5%)	20 (12.6%)	1 (4.5%)	83 (11.5%)
CABG	41 (9.1%)	50 (9.3%)	21 (13.2%)	1 (4.5%)	72 (10.0%)
Stroke/TIA	40 (8.9%)	52 (9.7%)	13 (8.2%)	2 (9.1%)	67 (9.3%)
Primary prevention	8 (1.8%)***	41 (7.6%)	10 (6.3%)	1 (4.5%)	52 (7.2%)
Arrhythmia/Rhythm device	21 (4.7%)	35 (6.5%)	14 (8.8%)	0 (0.0%)	49 (6.8%)
Heart failure	16 (3.5%)	22 (4.1%)	3 (1.9%)	1 (4.5%)	26 (3.6%)
Angina pectoris (stable/unstable)	25 (5.5%)	21 (3.9%)	3 (1.9%)	1 (4.5%)	25 (3.5%)
Cardiomyopathy	7 (1.6%)	17 (3.2%)	4 (2.5%)	3 (13.6%)	24 (3.3%)
MI	3 (0.7%)**	17 (3.2%)	4 (2.5%)	1 (4.5%)	22 (3.1%)
PVD	18 (4.0%)	13 (2.4%)	5 (3.1%)	1 (4.5%)	19 (2.6%)
SCAD	0 (0.0%)	3 (0.6%)	2 (1.3%)	0 (0.0%)	5 (0.7%)
Other	10 (2.2%)	20 (3.7%)	6 (3.8%)	3 (13.6%)	29 (4.0%)
Cardiovascular risk factors					
Hypertension	258 (57.0%)	314 (58.0%)	96 (60.4%)	13 (54.2%)	423 (58.4%)
Family history	193 (42.7%)***	300 (55.7%)‡	70 (43.8%)‡	14 (58.3%)	384 (53.1%)†
Dyslipidemia	241 (53.1%)	273 (50.7%)	77 (48.1%)	12 (50.0%)	362 (50.1%)
Diabetes	116 (25.6%)	128 (23.7%)	46 (28.9%)	6 (25.0%)	180 (24.9%)
Comorbidities					
Sleep apnea	91 (20.0%)*	76 (14.0%)	19 (11.9%)	4 (16.7%)	99 (13.6%)
Osteoarthritis	24 (5.3%)***	72 (13.3%)	26 (16.3%)	6 (25.0%)	104 (14.3%)
Cancer	24 (5.3%)**	56 (10.3%)	15 (9.4%)	3 (12.5%)	74 (10.2%)

N (%) or mean ± standard deviation shown

CABG = coronary artery bypass graft; CAD = coronary artery disease; CR = cardiac rehabilitation; MI = myocardial infarction; N = sample size; PCI = percutaneous coronary intervention; PVD = peripheral vascular disease; SCAD = spontaneous coronary artery dissection; TIA = transient ischemic attack

*Mann–Whitney U or chi-square test for difference between men and women in mixed-sex model: *P < .05, **P < .01, ***P < .001

^†^Kruskal–Wallis or chi-square test for difference between model among women: †*P* < .05; ††*P* < .01; †††*P* < .001

^‡^Post-hoc test results, where above significant: ‡*P* < .05; ‡‡*P* < .01; ‡‡‡*P* < .001

^§^Main referral event or procedure for each participant; in women, comparisons were only between supervised models due to low sample size in home-based

With regard to differences in outcomes at baseline in women in the supervised programs (Table 3), cardiorespiratory fitness was significantly higher in women in mixed-sex compared to those in women-only (*P* = 0.007 and *P* = 0.006 for VO_2peak_ and METs, respectively). No other differences were observed, however caution is warranted in interpreting the home-based data given the small sample size of retained participants.

Compared to the matched sample of men (Table 1), significantly less women in the supervised mixed-sex model were married or in a common-law relationship (*P* < 0.001), and lived with a spouse or partner (*P* < 0.001). In terms of clinical characteristics, PCI was a more common referral procedure in men (*P* < 0.001), while valvular heart disease (*P* = 0.015), primary prevention (*P* < 0.001), and myocardial infarction (*P* = 0.004) were more common in women (*P* < 0.001); family history was a more common CVD risk factor in women than men (*P* < 0.001). As for comorbidities, the proportion of sleep apnea in men was significantly higher than in women (*P* = 0.022), while the proportions of osteoarthritis and cancer were higher in women compared to men (*P* < 0.001 and *P* = 0.003, respectively). No other differences were observed (as per matching, there were no differences in age or CABG).

Compared to matched men pre-CR, cardiorespiratory fitness (*P* < 0.001 for both VO_2peak_ and METs) were significantly lower in women, while total cholesterol (*P* = 0.005), triglycerides (*P* = 0.007), HDL-C (*P* < 0.001), and depressive symptoms (*P* < 0.001) were significantly higher in women (Table 4).

### Outcomes

Differences in sociodemographic and clinical characteristics of those women who completed outcome assessments versus those who did not are shown in Table 2. As shown, with regard to sociodemographic characteristics, the proportion of retained female participants who spoke English as their first language was significantly greater than among those who dropped out. With regard to clinical characteristics, the proportion of retained female participants who had dyslipidemia was significantly greater than among those who dropped out. No other differences were observed. In matched men, compared to those lost to follow-up, there was a higher proportion of men with CABG among those who completed CR (n = 32, 11.3% vs. n = 9, 5.3%; *P* = 0.031), while the proportion of men with arrhythmia/rhythm device (n = 14, 8.3% vs n = 7, 2.5%; *P* = 0.005) was higher among those who dropped out than those who completed. No other differences were observed (data not shown).

**Table 2:** Women’s pre-CR characteristics by retention status, N = 727

	Retained/Completed N = 428 (58.9%)	Lost to follow-up/Dropouts N = 299 (41.1%)	*P* value*
*Sociodemographic*			
Age	67.5 ± 11.7	65.9 ± 13.1	.148
Marital status (% married/common-law)	201 (68.4)	132 (64.1)	.317
Highest educational attainment (% ≥ high school)	247 (94.3)	143 (90.5)	.146
Language spoken (% English)	328 (98.2)	214 (94.7)	.021
Occupational status			.067
Retired/no formal employment	185 (66.3)	115 (68.9)	–
Full-time/part-time/modified/restricted duties	84 (30.1)	39 (23.4)	–
Other (e.g., disability)	10 (3.6)	13 (7.8)	–
Travel time to CR centre (% 0–30 min)	104 (56.5)	75 (66.4)	.092
Living situation			.229
With spouse/partner	165 (52.4)	91 (46.2)	–
Alone	100 (31.7)	64 (32.5)	–
With family/friends/others	50 (15.9)	42 (21.3)	–
*Clinical*			
Referral event/procedure§			.217
PCI	158 (37.3)	88 (29.8)	–
Valvular heart disease	49 (11.6)	34 (11.5)	–
CABG	45 (10.6)	27 (9.2)	–
Stroke/TIA	39 (9.2)	28 (9.5)	–
Primary prevention	30 (7.1)	22 (7.5)	–
Arrhythmia/Rhythm device	25 (5.9)	24 (8.1)	–
Heart failure	9 (2.1)	17 (5.8)	–
Angina pectoris (stable/unstable)	12 (2.8)	13 (4.4)	–
Cardiomyopathy	13 (3.1)	11 (3.7)	–
MI	16 (3.8)	6 (2.0)	–
PVD	11 (2.6)	8 (2.7)	–
SCAD	3 (0.7)	2 (0.7)	–
Other	14 (3.3)	15 (5.1)	–
Cardiovascular risk factors			
Hypertension	251 (58.9)	172 (57.7)	.747
Family history of CVD	237 (55.9)	147 (49.2)	.074
Dyslipidemia	229 (54.0)	133 (44.6)	.013
Diabetes	95 (22.3)	85 (28.6)	.053
Comorbidities			
Sleep apnea	50 (11.7)	49 (16.4)	.069
Osteoarthritis	65 (15.2)	39 (13.0)	.417
Cancer	42 (9.8)	32 (10.7)	.696

N (%) or mean ± standard deviation shown

CABG = coronary artery bypass graft; CAD = coronary artery disease; CR = cardiac rehabilitation; MI = myocardial infarction; N = sample size; PCI = percutaneous coronary intervention; PVD = peripheral vascular disease; SCAD = spontaneous coronary artery dissection; TIA = transient ischemic attack

*Mann–Whitney U or chi-square test for difference between retained/completed and lost to follow-up/dropouts

^§^Main referral event or procedure for each participant

Table 3 displays outcome scores by model at each assessment point. Tobacco use was negligible. Post-program, among completers, 311 (90.4%) and 339 (98.5%) women had SBP and DBP under the guideline-recommended target of 140/90 mmHg, respectively; 160 (52.3%) women reached the guideline-recommended target for LDL-C of 1.8 mmol/L, and 195 (54.9%) women had a waist circumference of ≤ 88 cm. In terms of depressive symptoms, 207 (77.8%) women did not have elevated CES-D scores. These proportions did not differ significantly by model among women, but again caution is warranted in interpreting the home-based data given the small sample of retained participants.

**Table 3:** Outcomes by time and model, in women completers

	Mixed-sex N = 324			Women-only N = 96			Between Group†	Home-based N = 8		
	Intake	Discharge	Change*	Intake	Discharge	Change*	P	Intake	Discharge	Change*
Cardiorespiratory fitness										
VO_2peak_ (ml/kg/min)°	17.7 ± 5.3	20.8 ± 6.0	2.8 ± 2.7***	16.0 ± 4.5	19.8 ± 4.5	3.0 ± 2.7***	.008	18.3 ± 6.4	18.6 ± 6.2	0.6 ± 2.0
METs	5.1 ± 1.5	5.9 ± 1.7	0.8 ± 0.8***	4.6 ± 1.3	5.6 ± 1.3	0.9 ± 0.8***	.005	5.2 ± 1.8	5.2 ± 1.8	0.0 ± 0.7
Cardiovascular risk factors										
SBP (mmHg)	121.6 ± 17.6	120.3 ± 15.6	− 0.8 ± 17.1	124.0 ± 17.0	123.9 ± 15.1	1.9 ± 16.7	.068	123.9 ± 20.3	116.6 ± 17.5	− 5.4 ± 12.3
DBP (mmHg)	72.9 ± 9.8	73.1 ± 8.0	0.2 ± 9.4	72.2 ± 8.0	74.2 ± 8.0	1.9 ± 9.4	.997	67.7 ± 8.8	66.8 ± 6.6	− 1.7 ± 10.8
Total cholesterol (mmol/L)	4.1 ± 1.1	4.1 ± 1.2	− 0.0 ± 0.8	4.0 ± 1.2	4.0 ± 1.1	− 0.0 ± 0.6	.827	4.0 ± 1.3	5.0 ± 1.2	0.5 ± 1.3
Triglycerides (mmol/L)	1.3 ± 0.7	1.3 ± 0.7	− 0.0 ± 0.6	1.3 ± 0.8	1.3 ± 0.7	− 0.1 ± 0.4	.899	1.0 ± 0.3	1.5 ± 0.8	0.4 ± 0.3
HDL-C (mmol/L)	1.5 ± 0.4	1.5 ± 0.4	0.1 ± 0.2**	1.6 ± 0.5	1.5 ± 0.4	− 0.0 ± 0.0	.309	1.5 ± 0.5	1.2 ± 0.1	0.1 ± 0.2
LDL-C (mmol/L)	2.0 ± 1.0	2.0 ± 1.0	− 0.1 ± 0.6	2.0 ± 1.0	1.9 ± 0.9	0.0 ± 0.1	.752	2.1 ± 1.0	3.2 ± 0.7	0.2 ± 1.0
Tobacco use, (% current)	3 (5.9%)	2 (5.1%)	− 1 (2.7%)	0 (0.0%)	0 (0.0%)	0	N/A	0 (0.0%)	0 (0.0%)	0
Waist circumference (cm)	89.2 ± 13.9	87.9 ± 13.9	− 1.3 ± 6.3**	90.3 ± 13.5	89.2 ± 14.5	− 0.0 ± 0.7	.304	95.9 ± 17.6	92.6 ± 23.2	− 3.1 ± 7.2
BMI (kg/m^2^)	27.5 ± 5.7	27.3 ± 5.6	− 0.1 ± 1.4	27.8 ± 5.2	27.8 ± 5.9	0.0 ± 0.2	.524	31.9 ± 11.0	30.3 ± 11.9	− 1.6 ± 2.1
Psychosocial well-being										
Depressive symptoms (CES-D)	12.6 ± 9.9	10.4 ± 8.7	− 1.5 ± 8.1*	13.8 ± 8.5	11.2 ± 10.7	− 1.4 ± 2.3	.468	11.0 ± 6.8	13.0 ± 4.4	2.5 ± 0.7
Quality of life (Cantril’s ladder of life; 0–10)	6.8 ± 1.9	7.5 ± 1.5	0.7 ± 1.7**	7.2 ± 1.6	7.7 ± 1.6	0.3 ± 0.3	.157	7.0§	7.0§	0§§

N (%) or mean ± standard deviation shown for available sample at given assessment point; change score shown only in participants with scores at both time points

BMI = body mass index; CES-D = Center for Epidemiologic Studies-Depression scale; DBP = diastolic blood pressure; HDL-C = high-density lipoprotein cholesterol; LDL-C = low-density lipoprotein cholesterol; METs = metabolic equivalent of task; N = sample size; N/A = not applicable; SBP = systolic blood pressure; VO_2peak_ = maximum rate of oxygen consumption measured during cardiopulmonary exercise test

°Available in n = 285 (66.6%) patients

*Paired-samples t-test for change from intake to discharge within each CR model; *P < .05; **P < .01; ***P < .001. Tests in home-based group likely under-powered and therefore caution is warranted in interpreting those results

^†^Compared between mixed-sex and women-only only, as valid N in home-based is too low for some outcomes. General linear mixed model adjusts for baseline value

^‡^Baseline difference between mixed-sex and women-only: ‡*P* < .05; ‡‡*P* < .01; ‡‡‡*P* < .001

^§^Sample size = 1, standard deviation not available

^§§^No valid pair available for change score

As also shown in Table 3, among women, cardiorespiratory fitness significantly improved with mixed-sex and women-only CR (supervised models). With regard to cardiovascular risk factors, HDL-C and waist circumference significantly improved in mixed-sex. With regard to psychosocial outcomes, depressive symptoms and quality of life significantly improved in mixed-sex. No other changes were observed; there was low power for home-based, but what would be considered a clinically-significant reduction in SBP was observed.

As also shown in Table 3, between-group differences in women were assessed, but the home-based participants were excluded due to the small sample size post-program. Results showed that after adjusting for intake values, cardiorespiratory fitness was significantly greater with mixed-sex than women-only CR (for VO_2peak_: estimate = 1.67, standard error [SE] = 0.63, 95% Confidence Interval [CI] 0.43 to 2.91; for METS: estimate = 0.51, SE = 0.18, 95% CI 0.15 to 0.86). There were no significant differences in other clinical outcomes post-CR by supervised model; there was a trend favoring mixed-sex for SBP. Visual inspection of the home-based data suggests cardiorespiratory fitness, blood pressure, and HDL-C may be lower at discharge, while cholesterol, waist circumference and BMI higher than in the supervised models.

When examining sex differences in outcomes in the supervised mixed-sex program (Tables 3, 4), general linear mixed models adjusting for baseline values revealed cardiorespiratory fitness (*P* < 0.001 for both VO_2peak_ and METs), and quality of life (*P* = 0.002) were significantly lower in women, while triglycerides (*P* = 0.014), HDL-C (*P* < 0.001), LDL-C (*P* < 0.001), and depressive symptoms (*P* < 0.001) were significantly higher in women compared to men.

**Table 4:** Outcomes by time in matched, completing men in mixed-sex model, and difference from women in mixed-sex model

	Men N = 283			Intake sex difference† N = 270	Sex difference over 6 months‡		
	Intake	Discharge	Change*		Estimate	SE	95% CI (Lower, Upper)
Cardiorespiratory fitness							
VO_2peak_ (ml/kg/min)	21.8 ± 6.6	26.4 ± 8.2	4.4 ± 4.7***	− 4.5 ± 7.2***	− 4.80***	0.35	− 5.49, − 4.12
METs	6.3 ± 1.9	7.5 ± 2.4	1.2 ± 1.4***	− 1.4 ± 2.1***	− 1.39***	0.10	− 1.58, − 1.19
Cardiovascular risk factors							
SBP (mmHg)	119.5 ± 16.2	120.6 ± 16.2	1.2 ± 17.1	0.9 ± 23.0	0.37	0.97	− 1.53, 2.28
DBP (mmHg)	72.1 ± 10.5	72.7 ± 9.2	0.3 ± 10.7	1.1 ± 14.6	0.77	0.61	− 0.43, 1.97
Total cholesterol (mmol/L)	3.4 ± 1.0	3.5 ± 0.9	0.1 ± 0.7	0.5 ± 2.5**	0.67	0.07	0.54, 0.80
Triglycerides (mmol/L)	1.3 ± 0.7	1.2 ± 0.6	− 0.1 ± 0.5	0.2 ± 1.0**	0.11*	0.05	0.02, 0.20
HDL-C (mmol/L)	1.2 ± 0.3	1.3 ± 0.3	0.1 ± 0.2***	0.3 ± 0.5***	0.28***	0.24	0.23, 0.33
LDL-C (mmol/L)	1.7 ± 0.8	1.7 ± 0.7	− 0.0 ± 0.6	0.2 ± 2.4	0.33***	0.05	0.22, 0.43
Tobacco use, (% current)	5 (10.6%)	3 (10.3%)	− 2 (7.1%)	3 (3.3%)	N/A	N/A	N/A
Waist circumference (cm)	97.2 ± 12.1	95.8 ± 12.5	− 1.5 ± 5.1***	N/A	N/A	N/A	N/A
BMI (kg/m^2^)	27.6 ± 4.5	27.4 ± 4.7	− 0.3 ± 1.5**	0.4 ± 7.8	− 0.21	0.31	− 0.81, 0.40
Psychosocial well-being							
Depressive symptoms (CES-D)	9.7 ± 8.1	8.2 ± 7.5	− 1.7 ± 6.0**	4.8 ± 12.9***	3.39***	0.63	2.16, 4.63
Quality of life (Cantril’s ladder of life)	7.1 ± 1.7	7.4 ± 1.5	0.6 ± 1.5**	− 0.4 ± 2.5	− 0.48**	0.16	− 0.79, − 0.18

N (%) or mean ± standard deviation shown

BMI = body mass index; CES-D = Center for Epidemiologic Studies-Depression scale; CI = confidence interval; DBP = diastolic blood pressure; HDL-C = high-density lipoprotein cholesterol; LDL-C = low-density lipoprotein cholesterol; METs = metabolic equivalent of task; N = sample size; N/A = not applicable (i.e., sample size too small to run general linear mixed model, or not appropriate to test for sex differences in waist circumference due to biological differences); SBP = systolic blood pressure; SE = standard error; VO_2peak_ = maximum rate of oxygen consumption measured during cardiopulmonary exercise test

*Paired-samples t-test for change from intake to discharge in men; *P < .05; **P < .01; ***P < .001

^†^Paired-samples t-test for baseline difference between women and men (matched), men as reference category

^‡^General linear mixed model for sex difference; estimates for women with men as reference category

## Discussion

This novel study has examined women’s outcomes in all available CR models. While caution is warranted due to generalizability limits, the small sample of women completing the home-based model, and because the women-only program was not gender-tailored per se [23], contrary to hypotheses, the benefit of women-only CR was not evident. Outcome data suggested supervised CR resulted in greater cardiorespiratory fitness, with significant improvements from pre to post-program in risk factors and psychosocial well-being in mixed-sex participants only.

### Outcomes

An improvement of 1 ml/kg/min in VO_2peak_ is associated with 9–15% risk reduction in cardiac and overall mortality, both in men and women, and a 0.5 MET increase is associated with significantly lower mortality [42–45]. In this study, the improvement in cardiorespiratory fitness was clinically-meaningful in women in mixed-sex and women-only CR (i.e., the supervised models), but not in home-based (although caution is warranted due to the small sample size).

The somewhat greater utilization rates in the mixed-sex likely translated to the significantly greater functional capacity in women attending that model, although the higher fitness at intake likely also played a role; there was also a trend towards lower SBP with mixed-sex compared to women-only. Moreover, the greater utilization may have resulted in the significant increase in HDL-C and quality of life as well as reduction in depressive symptoms with mixed-sex, which were not observed with women-only. It was unexpected that the women-only model did not result in improved psychosocial well-being as has been observed with the 2 women-only CR RCTs [46–48]. Overall, clearly women-only did not result in better outcomes in this study as hypothesized.

### Sex differences

Consistent with literature [49, 50], women did come to CR with a poorer clinical profile than men. Women presented with poorer functional capacity, and had a poorer risk factor profile, but then they do have more to gain. Also consistent with literature, they were older, had less spousal support, different cardiac indications (i.e., women treated less aggressively), and more comorbidities [51]. Both women and men in supervised CR achieved significant improvements in cardiorespiratory fitness, again an indicator closely associated with mortality [45], but this was greater in men as shown in the literature. Quality of life was also better in men, with also lower lipids and depressive symptoms, even with adjustment for intake values. It is disheartening that we still have not closed this chasm in men’s and women’s outcomes, and based on this paper, women-only CR does not appear to be the answer we hoped it to be.

### Implications and research directions

The results of this study suggest there might not be enough benefit of a women-only model that is not gender-tailored to warrant the resources needed; indeed there is limited controlled research in this area and hence not a large body of high-quality evidence to inform policy and CR care provision decisions at the program-level [23, 28]. Given the limited sample and generalizability, it is premature to draw conclusions regarding whether women-only CR can address gender bias in CVD secondary prevention. Further research should consider whether offering some tailored women-only sessions might be engaging and cost-efficient [22]. Perhaps we should also be better at standardizing what gender-tailoring is desired by women in practice as well as what can lead to increased program engagement, and subsequently then better outcomes.

Despite the many constraints women have to coming on-site, results of this study also suggest we need to question and investigate whether women adhere and push themselves to achieve outcome improvements in unsupervised settings to the same degree. With such little post-program data in this cohort this is difficult to test however. More research is needed, preferably with a randomized design, comparing these outcomes in women participating in supervised and unsupervised models.

### Limitations

Caution is warranted when interpreting these findings due to several study limitations. Chiefly, generalizability is limited due to the fact that the study was conducted at one academic health science centre, so the women-only offerings may be different than at other centres. Moreover, the CR programs in this study offer a fairly high dose of CR compared to other jurisdictions [22], which may impact outcomes. Finally, most women-only programs are offered in the Eastern Mediterranean region [22]; it is likely these results are not generalizable to that setting.

Second, there was some retention bias for the third objective, approximately 40% of participants did not attend their discharge assessment, and this did vary by program model [16, 41]. Women’s high rate of CR dropout is well-documented in the literature. Third, the sample size in home-based was small, and this is compounded by the low retention mentioned above, rendering analyses likely under-powered. Hence, some outcome changes in the third objective could not be assessed due to the lack of available data; there may be differences not identified by this study, so more research is warranted.

Fourth, multiple comparisons were performed, which can increase error rates. Fifth, there are challenges associated with using administrative data; missing and implausible values were checked against hard charts at the beginning, but due to COVID-19 we could not check all values. Sixth, with regard to measurement, best practices in blood pressure assessment were not followed, and thus there is likely some error. Seventh, individual exercise prescriptions were not compared between models, and therefore superiority of the mixed-sex model due to greater exercise dose cannot be ruled out.

Finally, the study design was not randomized. Causal conclusions cannot be drawn. Furthermore, participants electing women-only CR were more often not in formal employment and were less likely to have a family history of CVD than those choosing mixed-sex CR. There were no significant differences for many other sociodemographic and clinical characteristics, but again due to design, the impact of selection bias on outcomes cannot be known.

## Conclusion

Participants in mixed-sex only achieved significant improvements in HDL-C, waist circumference, quality of life, and depressive symptoms by program end; Fitness was significantly greater with mixed-sex than women-only. The study was under-powered to make comparisons to home-based, but improvements in cardiorespiratory fitness require more study. Whether fully gender-tailored programs are advantageous requires more controlled, large-scale investigation. We need to better engage women in all models of CR, to ensure they achieve optimal outcomes.

## Data Availability

Due to the nature of this research, participants of this study did not agree for their data to be shared publicly, so supporting data cannot not be made publicly available. Data are available upon request to the corresponding author by qualified investigators with appropriate approvals.

## Abbreviations

CR: Cardiac rehabilitation
VO_2_: Volume of oxygen consumed per unit of time
CVD: Cardiovascular disease
PCI: Percutaneous coronary intervention
CABG: Coronary artery bypass surgery
UHN: University Health Network
CAD: Coronary artery disease
METs: Metabolic equivalent of task
SBP: Systolic blood pressure
DBP: Diastolic blood pressure
LDL-C: Low density lipoprotein cholesterol
CES-D: Centre for epidemiologic studies-depression scale
SD: Standard deviation
HDL-C: High density lipoprotein cholesterol
BMI: Body mass index
RCT: Randomized controlled trial
